# A multi-country cross-sectional study of self-reported sexually transmitted infections among sexually active men in sub-Saharan Africa

**DOI:** 10.1186/s12889-020-09996-5

**Published:** 2020-12-07

**Authors:** Abdul-Aziz Seidu, Bright Opoku Ahinkorah, Louis Kobina Dadzie, Justice Kanor Tetteh, Ebenezer Agbaglo, Joshua Okyere, Tarif Salihu, Kenneth Fosu Oteng, Eustace Bugase, Sampson Aboagye Osei, John Elvis Hagan, Thomas Schack

**Affiliations:** 1 Department of Population and Health, University of Cape Coast, Cape Coast, Ghana; 2 College of Public Health, Medical and Veterinary Sciences, James Cook University, Townsville, Queensland Australia; 3 School of Public Health, Faculty of Health, University of Technology Sydney, Ultimo, Australia; 4 Department of English, University of Cape Coast, Cape Coast, Ghana; 5 Ashanti Regional Health Directorate, Ghana Health Service, Kumasi, Ghana; 6 Department of Health Policy Planning and Management, School of Public Health, University of Ghana, Accra, Ghana; 7 Department of Geography and Regional Planning, University of Cape Coast, Cape Coast, Ghana; 8 Department of Health, Physical Education, and Recreation, University of Cape Coast, Cape Coast, Ghana; 9 Neurocognition and Action-Biomechanics-Research Group, Faculty of Psychology and Sport Sciences, Bielefeld University, Bielefeld, Germany

**Keywords:** DHS, Global Health, Men, Public health, STIs, Sub-Saharan Africa

## Abstract

**Background:**

Despite the importance of self-reporting health in sexually transmitted infections (STIs) control, studies on self-reported sexually transmitted infections (SR-STIs) are scanty, especially in sub-Saharan Africa (SSA). This study assessed the prevalence and factors associated with SR-STIs among sexually active men (SAM) in SSA.

**Methods:**

Analysis was done based on the current Demographic and Health Survey of 27 countries in SSA conducted between 2010 and 2018. A total of 130,916 SAM were included in the analysis. The outcome variable was SR-STI. Descriptive and inferential statistics were performed with a statistical significance set at *p* < 0.05.

**Results:**

On the average, the prevalence of STIs among SAM in SSA was 3.8%, which ranged from 13.5% in Liberia to 0.4% in Niger. Sexually-active men aged 25–34 (AOR = 1.77, CI:1.6–1.95) were more likely to report STIs, compared to those aged 45 or more years. Respondents who were working (AOR = 1.24, CI: 1.12–1.38) and those who had their first sex at ages below 20 (AOR = 1.20, CI:1.11–1.29) were more likely to report STIs, compared to those who were not working and those who had their first sex when they were 20 years and above. Also, SAM who were not using condom had higher odds of STIs (AOR = 1.35, CI: 1.25–1.46), compared to those who were using condom. Further, SAM with no comprehensive HIV and AIDS knowledge had higher odds (AOR = 1.43, CI: 1.08–1.22) of STIs, compared to those who reported to have HIV/AIDS knowledge. Conversely, the odds of reporting STIs was lower among residents of rural areas (AOR = 0.93, CI: 0.88–0.99) compared to their counterparts in urban areas, respondents who had no other sexual partner (AOR = 0.32, CI: 0.29–0.35) compared to those who had 2 or more sexual partners excluding their spouses, those who reported not paying for sex (AOR = 0.55, CI: 0.51–0.59) compared to those who paid for sex, and those who did not read newspapers (AOR = 0.93, CI: 0.86–0.99) compared to those who read.

**Conclusion:**

STIs prevalence across the selected countries in SSA showed distinct cross-country variations. Current findings suggest that STIs intervention priorities must be given across countries with high prevalence. Several socio-demographic factors predicted SR-STIs. To reduce the prevalence of STIs among SAM in SSA, it is prudent to take these factors (e.g., age, condom use, employment status, HIV/AIDS knowledge) into consideration when planning health education and STIs prevention strategies among SAM.

## Background

Sexually transmitted infections (STIs) are a cohort of infections that are transmitted through sexual intercourse [1]. Notable amongst these infections are gonorrhea, syphilis, trichomoniasis, and chlamydia [2]. Although majority of these STIs are curable, their implications on health and wellbeing cannot be underestimated. Severe forms of STIs may result in some complications including blindness, infertility, cardiovascular diseases, and higher risk of HIV acquisition [1, 3].

Amidst the seemingly preventive strategies for STIs and the relatively cost-effective and simple treatments, STI prevalence continues to be high. Reports from the WHO show that in 2012, there was about 367 million new cases of curable STIs [4]. These infections are pervasive in Asia, sub-Saharan Africa (SSA), and Latin America [4]. SSA alone contributes 93 million cases of STIs per annum [2].

Given the public health impact of STIs against the fact that most of the infections are curable, the WHO Global Health Sector Strategy on Sexually Transmitted Infections 2016–2021 laid out a roadmap for STI prevention and control [5]. The first step in this roadmap is directed at the collection of data on STI incidence and its prevalence [6]. To achieve this objective, individuals who contract STIs must self-report it for data capturing and subsequent treatment. Self-reporting is a system through which a medical history of a patient is queried in order to ascertain an individual’s risk status and/or assess the prevalence of diseases in a particular population [7–9]. Therefore, studies that account for self-reported STIs (SR-STIs) are essential for public health and policy interventions.

Despite the importance of self-reporting health in STI control, research on SR-STI has received less attention. There is limited evidence on SR-STI among males in SSA. Extant literature has predominantly focused on HIV partly due to its incurability and wide spread. Other studies have focused on minority groups such as men who have sex with men (MSM) and the aged [10]. In SSA, there has not been any study that has been done on a large scale using nationally-representative survey data to examine the prevalence and factors associated associated with SR-STIs. Therefore, the current multi-country cross-sectional study examined the prevalence and factors associated with SR-STIs among sexually active men (SAM) in SSA. Findings from the study would be significant towards the design and implementation of STI strategies such as strengthening surveillance, program monitoring, early diagnosis, as well as men and partner management to meet SSA context-specific challenges.

## Methods

### Data source

We pooled data from the Demographic and Health Survey (DHS) of 27 countries in SSA conducted between 2010 and 2018, which had information on SR-STI (Table 1). Specifically, we used data from the men’s file from the various countries. The DHS is a nationally representative survey that is conducted in over 85 low- and middle-income countries globally through a two-stage stratified sampling protocol. The survey focuses on essential maternal and child health markers and men’s health, including SR-STIs [10]. The dataset is freely accessible via this link: https://dhsprogram.com/data/available-datasets.cfm. Details of the DHS methodology have been reported in previous studies [10, 11]. A sample of 130,196 men in SSA who had ever had sexual intercourse in the past 12 months and had complete information on all the variables of interest was used. The ‘Strengthening the Reporting of Observational Studies in Epidemiology’ (STROBE) statement was followed in conducting this research.

**Table 1:** Sample size of the study (weighted)

Country, Year of survey	Frequency	Percentage
1. Burkina Faso, 2010	5092	3.9
2. Benin, 2017–2018	5259	4.0
3. Burundi, 2016–2017	4555	3.5
4. DR Congo, 2013–2014	6578	5.1
5. Congo, 2011–2012	4292	3.3
6. Cote D’Ivoire, 2011–2012	3838	3.0
7. Cameroon, 2011	5223	4.0
8. Ethiopia, 2016	7981	6.1
9. Gabon, 2012	4541	3.5
10. Ghana, 2014	3101	2.4
11. Gambia, 2013	1990	1.5
12. Guinea, 2018	2145	1.7
13. Kenya, 2014	9562	7.3
14. Comoros, 2012	1350	1.0
15. Liberia, 2013	3290	2.5
16. Lesotho, 2014	1534	1.2
17. Mali, 2018	2385	1.8
18. Malawi, 2015–2016	5717	4.4
19. Mozambique, 2011	4436	3.4
20. Nigeria, 2018	10,777	8.3
21. Niger, 2012	2555	2.0
22. Namibia, 2013	3189	2.5
23. Sierra Leone, 2013	5454	4.2
24. Senegal, 2017	2583	2.0
25. Chad, 2014–2015	3056	2.4
26. Togo, 2013–2014	10,821	8.3
27. Zambia, 2018	5733	4.4
Total	130,196	100.0

### Study variables

#### Outcome variable

﻿The outcome variable in this analysis was STIs among SAM. It is a variable with a dichotomous outcome (Yes/No). Specifically, men were asked whether they had a disease they acquired through sexual contact in the past 12 months [1].

### Independent variables

Independent variables included in this analysis were age (15–24, 25–34, 35–44, 44+), residence (rural, urban), educational level (no education, primary, secondary/higher), wealth status (poor, middle, rich), marital status (married, not married), employment status (working, not working), age at first sex (<=19, 20+), number of sexual partners in the last 12 months excluding the spouse (0,1, 2+), comprehensive HIV and AIDS knowledge (Yes, No), HIV testing (Yes, No), exposure to mass media (newspaper, radio, TV) (Yes, No), and health insurance coverage (Yes, No) (see Table 2).

**Table 2:** Socio-demographic characteristics and self-reported STIs among sexually active men in sub-Saharan Africa (Weighted)

Variable	Frequency	Percentage	SR-STIs	
			No	Yes
**Age (*p*** **< 0.001)fe**				
15–24	27,436	21.1	95.2	4.8
25–34	42,592	32.7	95.4	4.6
35–44	34,548	26.5	96.8	3.2
45+	25,620	19.7	97.9	2.1
**Residence (*p*** **< 0.001)**				
Urban	53,352	41.0	95.6	4.4
Rural	76,844	59.0	96.7	3.3
**Educational level (*p*** **< 0.001)**				
No education	29,263	22.5	97.2	2.8
Primary	38,694	29.7	96.4	3.6
Secondary/higher	62,239	47.8	95.6	4.4
**Wealth (*p*** **= 0.857)**				
Poor	45,331	34.8	96.4	3.6
Middle	25,256	19.4	96.3	3.7
Rich	59,609	45.8	96.1	3.9
**Marital status (p < 0.001)**				
Not married	49,328	37.9	94.4	5.6
Married	80,868	62.1	97.3	2.7
**Occupation (*p*** **= 0.001)**				
Not working	11,470	8.8	95.4	4.6
Working	118,726	91.2	96.3	3.7
**Age at first sex (*p*** **< 0.001)**				
< =19	79,867	61.3	95.3	4.7
20+	50,329	38.7	97.6	2.4
**Number of sex partners excluding spouse in the last 12 months (*p*** **< 0.001)**				
0	82,434	63.3	97.6	2.4
1	36,759	28.2	95.2	4.8
2+	11,004	8.5	89.4	10.6
**Paid for sex (*p*** **< 0.001)**				
No	113,339	87.1	96.8	3.2
Yes	16,857	13.0	92.2	7.8
**Used condom during sex (*p*** **= 0.009)**				
No	102,040	78.4	96.3	3.7
Yes	28,156	21.6	95.9	4.1
**Comprehensive HIV and AIDS Knowledge (*p*** **< 0.001)**				
No	70,066	53.8	95.9	4.1
Yes	60,130	46.2	96.6	3.4
**Ever tested for HIV (*p*** **= 0.065)**				
No	71,876	55.2	96.1	3.9
Yes	58,320	44.8	96.3	3.7
**Frequency of reading newspapers (*p*** **< 0.001)**				
No	80,791	62.1	96.5	3.5
Yes	49,405	38.0	95.8	4.2
**Frequency of listening to radio (*p*** **= 0.910)**				
No	28,075	21.6	96.3	3.7
Yes	102,121	78.4	96.2	3.8
**Frequency of watching television (*p*** **= 0.960)**				
No	56,460	43.4	96.3	3.7
Yes	73,736	56.6	96.1	3.9
**Covered by health insurance (*p*** **= 0.044)**				
No	117,216	90.0	96.2	3.8
Yes	12,980	10.0	96.8	3.2

*P-values are from Chi-square Test

HIV testing was measured by asking the participants this question: ‘Have you ever tested for HIV?’. Exposure to mass media was captured as follows: “Do you watch television almost every day, at least once a week, less than once a week or not at all? ﻿Do you read a newspaper or magazine at least once a week, less than once a week or not at all? ﻿Do you listen to the radio at least once a week, less than once a week or not at all?” The responses included the following: Not at all, less than once a week, and at least once a week. The responses from these questions were then categorized as Yes/No.

Wealth, in the DHS, is a composite measure computed by combining data on a household’s ownership of carefully identified assets including television, bicycle, materials used for house construction, sanitation facilities, and type of water access. Principal component analysis was used to transform these variables into wealth index by placing individual households on a continuous measure of relative wealth. The DHS segregates households into five wealth quintiles: poorest, poorer, middle, richer, and richest. These parameters were then grouped into three: poorest, poorer (Poor), Middle and, richer and richest (rich).

Comprehensive HIV knowledge was defined as knowing that consistent use of condoms during sexual intercourse and having just one uninfected faithful partner can reduce the chance of getting AIDS virus, knowing that a healthy-looking person can have the AIDS virus, and rejecting the two most common local misconceptions about AIDS transmission or prevention (i.e., mosquito bites can give HIV and HIV can be gotten from witchcraft and supernatural means). Comprehensive HIV knowledge was coded as Yes = 1 and No = 0. These factors were chosen based on their theoretical and empirical relationship with SR-STIs in previous studies [1, 2].

### Statistical analyses

Stata version 14.0 was used to conduct the analyses. Both descriptive and inferential analyses were carried out. Descriptive statistics were calculated to characterize men. The data on men were weighted to account for sampling probability and non-response. Besides, the data were adjusted to account for the complex survey design and robust standard errors. Bivariate logistic regression analysis was conducted to select potential variables for the follow-up multivariable logistics regression analysis. Variables with a *p* < 0.05 in the bivariate analysis were included in the multivariable logistic regression model. Before fitting the final model, multi-collinearity between the independent variable was checked (Mean VIF = 1.35, Minimum = 1.05, Maximum VIF = 2.01) were deemed satisfactory. The multivariable binary logistic regression analysis was performed to identify factors associated with STIs. The reference categories were informed by previous studies and a priori. The descriptive results were presented as proportions while the regression results were presented as crude odds ratios (cORs) and adjusted odds ratios (aORs) with 95% confidence intervals and *p*-values. The statistical tests were reported as significant if *p*-value < 0.05 and the 95% confidence interval did not contain the null value.

## Results

### Socio-demographic characteristics and self-reported sexually transmitted infections among men in SSA

**Fig. 1:**
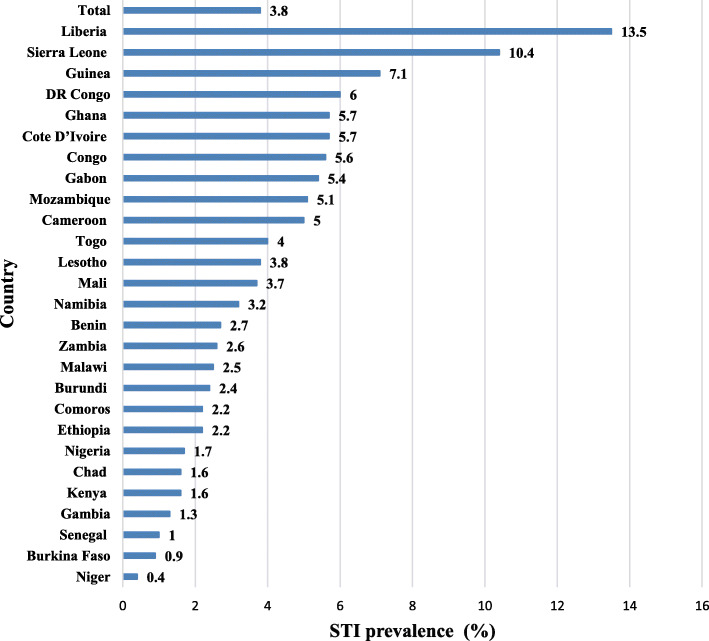
Prevalence of self-reported STIs among sexually active men in sub-Saharan Africa

Figure 1 shows the socio-demographic characteristics and prevalence of SR-STIs among SAM in the 27 countries in SSA. On the average, the prevalence of STIs among SAM in SSA was 3.8%, which ranged from 13.5% in Liberia to 0.4% in Niger.

### Socio-demographic characteristics and self-reported STIs among men in SSA (weighted)

Age, residence, educational level, marital status, occupation, age at first sex, number of sexual partners, paid sex, condom use during sex, comprehensive HIV and AIDS knowledge, frequency of reading newspapers, and health insurance coverage were found as having statistically significant associations with SR-STIs among SAM. Specifically, the highest prevalence of SR-STIs was found among those aged 15–24 (4.8%), urban residents (4.4%), respondents who had secondary or higher education (4.4%), those of the rich wealth quintile (3.9%), and never married SAM (5.6%). Similarly, prevalence of SR-STIs was higher among SAM who were not working (4.6%), those whose age at first sex was below 20 years (4.7%), those with 2 or more sexual partners excluding their spouses (10.6%), SAM who reported to have paid for sex (7.8%), those who had no comprehensive HIV and AIDS knowledge (4.1%), and SAM from the 27 countries in SSA who had health insurance coverage (3.2%) (see Table 2).

### Multivariable logistic regression analysis on the factors associated with STIs among men in SSA

Table 3 shows the results from the multivariable logistic regression analysis of the factors associated with STIs among SAM in SSA, after controlling for country in Model 2. Results showed that SAM aged 25–34 (AOR = 1.77, CI:1.6–1.95), 15–24 (AOR = 1.41, CI: 1.25–1.59), and 35–44 (AOR = 1.45, CI: 1.31–1.61) were more likely to report STIs, compared to those aged 45 or more years. SAM who were residents in rural areas were less likely to report STIs (AOR = 0.93, CI: 0.88–0.99), compared to their counterparts in urban areas. For occupation, SAM who were working had higher odds of STIs (AOR = 1.24, CI: 1.12–1.38), compared to those who were not working. Respondents who had their first sex at ages below 20 were also more likely to report STIs (AOR = 1.20, CI:1.11–1.29) compared to those who had their first sex when they were 20 years and above. SAM who had no other sexual partner (AOR = 0.32, CI: 0.29–0.35) had lower odds of STIs compared to those who had 2 or more sexual partners excluding their spouses. SAM who reported to have not paid for sex were less likely to report STIs (AOR = 0.55, CI: 0.51–0.59), compared to those who reported to have paid for sex. Also, SAM who were not using condom had higher odds of STIs (AOR = 1.35, CI: 1.25–1.46), compared to those who were using condom. Further, SAM with no comprehensive HIV and AIDS knowledge had higher odds (AOR = 1.43, CI: 1.08–1.22) of STIs, compared to those who reported to have the knowledge. SAM who did not read newspapers had lower odds (AOR = 0.93, CI: 0.86–0.99) of STIs, compared to SAM who read. Among the 27 participating countries, SAM in Liberia had the highest odds of STIs (AOR = 5.99, CI: 4.12–8.73) followed by Sierra Leone (AOR = 4.57, CI: 3.15–6.64). SAM in Burkina Faso recorded the lowest odds of STIs (AOR = 0.38, CI: 0.231–0.625) followed by Niger (AOR = 0.45, CI: 0.239–0.834) and Senegal (AOR = 0.47, CI: 0.273, 0.795).

**Table 3:** Binary logistic regression analysis on the factors associated with self-reported STIs among sexually active men in sub-Saharan Africa

Variable	Model I cOR[95%CI]	Model II aOR[95%CI]
**Age**		
15–24	2.382***[2.156,2.632]	1.414***[1.255,1.593]
25–34	2.200***[1.999,2.420]	1.768***[1.600,1.953]
35–44	1.549***[1.397,1.717]	1.449***[1.307,1.608]
45+	Ref	Ref
**Residence**		
Rural	0.808***[0.763,0.855]	0.930*[0.876,0.991]
Urban	Ref	Ref
**Educational level**		
Second/higher	1.574***[1.455,1.704]	1.008[0.912,1.114]
Primary	1.317***[1.207,1.436]	1.095[0.994,1.206]
No education	Ref	Ref
**Marital status**		
Not married	Ref	Ref
Married	0.487***[0.460,0.515]	0.971[0.890,1.058]
**Occupation**		
Working	0.857** [0.782,0.940]	1.242***[1.122,1.376]
Not working	Ref	Ref
**Age at first sex**		
< =19	2.052***[1.920,2.193]	1.196***[1.111,1.288]
20+	Ref	Ref
**Number of sex partners excluding spouse in the last 12 months**		
0	0.219***[0.203,0.235]	0.315***[0.285,0.349]
1	0.439***[0.407,0.473]	0.512***[0.474,0.552]
2+	Ref	Ref
**Paid for sex**		
No	0.405***[0.380,0.432]	0.548***[0.511,0.588]
Yes	Ref	Ref
**Condom usage**		
No	0.915** [0.856,0.978]	1.348***[1.245,1.459]
Yes	Ref	Ref
**Comprehensive HIV and AIDS knowledge**		
No	1.232***[1.163,1.305]	1.143***[1.075,1.216]
Yes	Ref	Ref
**Exposure to newspaper**		
No	0.881***[0.832,0.934]	0.926*[0.864,0.993]
Yes	Ref	Ref
**Health insurance coverage**		
No	1.105*[1.002,1.218]	1.098[0.983,1.227]
Yes	Ref	Ref
**Country**		
Burkina Faso	0.300***[0.182,0.493]	0.380***[0.231,0.625]
Benin	1.295[0.870,1.928]	1.237[0.831,1.842]
Burundi	1.072[0.710,1.618]	1.648*[1.090,2.491]
Dr. Congo	2.657***[1.821,3.877]	2.109***[1.445,3.077]
Congo	2.899***[1.976,4.252]	1.882**[1.280,2.765]
Cote D’ivoire	2.282***[1.543,3.375]	1.951***[1.320,2.885]
Cameroon	2.356***[1.606,3.456]	2.078***[1.417,3.048]
Ethiopia	0.686[0.457,1.029]	0.901[0.601,1.349]
Gabon	2.409***[1.638,3.542]	2.021***[1.368,2.983]
Ghana	2.669***[1.801,3.955]	2.964***[1.996,4.402]
Gambia	0.701[0.423,1.163]	0.894[0.539,1.483]
Guinea	3.309***[2.225,4.921]	3.729***[2.509,5.542]
Kenya	0.737[0.495,1.098]	0.799[0.537,1.189]
Liberia	6.665***[4.578,9.703]	5.996***[4.117,8.731]
Lesotho	1.401[0.879,2.235]	1.185[0.743,1.890]
Mali	1.677* [1.101,2.554]	2.241***[1.470,3.416]
Malawi	1.169[0.784,1.742]	1.171[0.786,1.743]
Mozambique	2.438***[1.657,3.589]	2.169***[1.474,3.193]
Nigeria	0.825[0.559,1.219]	0.935[0.634,1.379]
Niger	0.283***[0.152,0.528]	0.447*[0.239,0.834]
Namibia	1.738** [1.158,2.607]	2.028***[1.347,3.053]
Sierra Leone	4.839***[3.331,7.030]	4.571***[3.145,6.642]
Senegal	0.412** [0.241,0.704]	0.466**[0.273,0.795]
Chad	0.774[0.488,1.227]	0.928[0.586,1.470]
Togo	0.922[0.594,1.432]	1.076[0.694,1.670]
Zambia	1.876**[1.288,2.731]	1.879***[1.292,2.732]
Zimbabwe	1.307[0.881,1.939]	1.275[0.860,1.891]
Comoros	Ref	Ref
**N**	**130,196**	**130,196**

Exponentiated coefficients; 95% confidence intervals in brackets*p < 0.05,** *p* < 0.01, ***p < 0.001, *cOR* Crude Odds Ratio; *aOR* Adjusted Odds Ratios

## Discussion

### Summary of key findings

This study was conducted to assess the prevalence and factors associated with SR-STIs among SAM in SSA using the DHS data sampled from 27 countries. The study showed that the prevalence of SR-STIs among SAM in SSA was 4.4%. The factors associated with STIs were age, place of residence, occupation, age at first sex, number of sexual partners excluding spouse, paid sex, condom use, comprehensive HIV and AIDS knowledge, and exposure to newspapers.

### Synthesis with previous evidence

An average prevalence of 3.8% SR-STIs was found among the 27 sampled countries, a finding that corroborates previous studies [e.g., [1, 12]]. However, there were substantial variations of prevalence at the country level. SAM in Liberia had the highest odds of STIs, followed by Sierra Leone and Guinea. Plausible reasons for these higher odds of STIs in some of the countries could be as a result of variations in socio-cultural practices and beliefs that affected health-seeking behaviors towards STIs. For example, in Liberia, STI patients still resort to the use of traditional methods in treating their infections due to their traditional ethno-medical beliefs [13]. Ironically, Green’s [13] study in Liberia revealed that traditional healers in the country had little or no knowledge about STIs, including HIV/AIDS. Other countries that recorded higher odds of STIs among SAM were Zambia, Namibia, Mozambique, Mali, Ghana, Gabon, Cameroun, Cote D’Ivoire, Congo, DR Congo, and Burundi. In the current study, Burkina Faso had the lowest odds of STIs in the sub-region, followed by Niger and Senegal. As reported in Burkina Faso, good access to healthcare and widespread use of antibiotics for the treatment of STIs could account for this finding [14].

A statistically significant association between age and SR-STIs among SAM in SSA was also established in the current work. The odds of STIs were higher for SAM in the 15–44 age group than those in the 45+ age group. Supporting this finding, evidence from available literature shows that young men involve in much riskier sexual behaviors including paying for sex, compared to their older counterparts [15–19]. SAM in SSA who had their first sex below age 20 had higher odds of STIs than their counterparts who had their first sex after they were 20 years old. Several studies have reported similar findings in other countries: China [20, 21], Lesotho [22], and Republic of Korea [23]. The plausible reason could be the risky sexual behaviors through experimentation often noted with adolescents. Adolescents are known to engage in several risky sexual behaviors such as having multiple sexual partners, experimenting with sex, non-condom use, and alcohol use during sexual intercourse [18, 19, 24–28]. Such risky sexual behaviors during the transition period to adulthood are known to predispose sexually active men to all forms of STIs.

The study also found that SAM resident in rural areas had lower odds of STIs, compared to their counterparts who were resident in urban areas. Urban areas are sometimes filled with migrants from rural areas and the anonymity of being a foreigner might increase risky sexual activities such as multiple sexual partners, engaging in sex with commercial sex workers, and alcohol abuse [29]. The current finding supports a survey conducted in SSA that revealed paid sex as a high-risk factor for STIs. Seidu et al. [15] showed in their study that SAM in rural areas had lower odds to pay for sex, compared to men in urban areas. Other studies in Botswana [30], China [31], and Cambodia [32] have also reported that SAM in urban areas are more exposed to STIs due to their higher odds of paying for sex. Disparities in socio-economic and healthcare access between urban and rural areas in SSA could partly explain this trend. Also, men who are more capable and willing to pay for sex are in the urban areas where commercial sex workers usually operate [33]. This finding also supports another finding in the current study where men who reported to have not paid for sex were less likely to report STIs, compared to those who reported to have paid for sex.

Comprehensive HIV and AIDS knowledge was significant in predicting the odds of contracting STIs in SSA. SAM with no comprehensive HIV and AIDS knowledge had higher odds of STIs, compared to those who reported to have the knowledge. A similar finding has been reported from a comparative cross-sectional study in Ethiopia, where participants with comprehensive HIV/AIDS knowledge had fewer sexual partners, used condoms, and reported fewer incidents of STIs, as compared to their comparative group with less HIV/AIDS knowledge who reported having multiple sexual partners, non-condom use, and many reporting STIs [34]. Just like other STIs, HIV can be transmitted through sexual intercourse. Therefore, comprehensive knowledge of HIV/AIDS and its transmission would help SAM in SSA to make informed decisions such as the use of condoms, reduction of sexual partners, and practicing safer sex. A significant association between sexual attitude and knowledge has been reported in other studies (e.g., in India, [35]). Similarly, a significant association between condom use and the odds of STIs has been reported in Ghana [36] and in Europe [37]. SAM who were not using condom had higher odds of STIs, compared to those who were using condom. Condom use is known to effectively reduce risk of STIs, including HIV/AIDS, when they are used properly and consistently [4].

Other findings that SAM who had no sexual partner apart from their spouse and those with only one sexual partner had lower odds of STIs, compared to those who had 2 or more sexual partners excluding their spouses. Similar studies in Ethiopia [1, 38], South Africa [5], Kenya [39], and Lao PDR [40] have all documented a significant association between number of sexual partners and odds of STIs. Having multiple sexual partners is a high-risk sexual behavior for STIs [41, 42]. Considering that the awareness on STIs including HIV testing is very low in SSA [43], people could be carrying infections without knowing. Therefore, multiple sexual partners will only increase one’s chances of being infected.

In contrast to some studies [1, 44, 45], other results revealed that SAM who did not read newspapers had lower odds of STIs, compared to those who read newspapers. Seidu et al. [15] reported that men who had exposure to newspaper had higher odds of paying for sex, a risky sexual behavior for STIs. Some newspapers may carry some sexually explicit contents which could entice SAM to engage in risky sexual behaviors. Newspapers are easily accessible in urban areas compared to the rural areas. This trend implies that urban dwellers who may readily access these newspapers would have higher odds of STIs. Asekun-Olarinmoye et al. [46] indicated in a study conducted in Nigeria that exposure to mass media could trigger risky sexual behaviors by serving as risk factors for STIs.

### Practical implications

A population-based demographic and behavioural study like this offers useful information for planning and evaluating STI prevention and control programmes of low- to hig-risk populations across selected SSA countries. STIs prevalence across selected SSA showed distinct cross-country variations. Current findings suggest that STI interventions priorities must be given across countries with high prevalence. For the noted high-risk populations (e.g., Liberia, Sierra Leone, Guinea), country-level STI referral centres, laboratories, surveillance systems, and human resource capacity ought to be strengthened. Current findings imply that when looking at STI prevalence and perhaps incident infections among SAM for appropriate interventions, considerations must be given to persons with socio-economic disparities (i.e., low, -middle, and upper- income profile), aged 15 to 44, and within urban settlements. Behavioural programmes (e.g., abstinence and condom initiatives) ought to consider some of the socio-cultural orientations (e.g., patriarchal norms) of SAM in SSA. Having multiple sexual partners has been well documented as a gateway for STI infections. Therefore, advocacy on abstinence and being faithful to one sexual partner are effective strategies to prevent STIs among SAM across studied countries in SSA. Media outlets in high-risk populations could have their programme contents regulated by their national media communication authorities, especially on programmes that might promote illicit sexual behaviours or practices among their sexually active population. The approach might reduce the proliferation of information (e.g., widespread use of aphrodisiac) that promotes unsafe sexual practices among SAM against STIs.

### Strength and limitations

Nationally representative data were used to assess the factors associated with STIs among SAM in SSA. The data collection technique and methodology employed followed best practices by experienced and well-trained data collectors, which resulted in a high response rate. The current findings can, therefore, be generalized to all SAM in SSA, and the rigorous analyses used make the findings credible and valid. Despite these strengths, causal interpretation cannot be deduced from the study findings due to the cross-sectional study design employed. The outcome variable was also measured based on self-reports which were not validated by any medical practitioner through testing. Hence, information provided might be subject to some biases. Also, study participants did not indicate the exact type of STI.

## Conclusion

The study showed that the prevalence of SR-STIs among SAM in SSA was 4.4%. To tackle STIs among SAM in SSA, it is prudent to take the factors associated with SR-STI into consideration when planning health education and STIs prevention strategies. Specifically, health education interventions should focus on educating men about STIs including HIV/AIDS and sensitize men on the need to reduce the number of sexual partners. These can be done by paying attention to the demographic and socio-economic differences among men.

## Data Availability

The dataset can be accessed at https:// https://dhsprogram.com/data/dataset/

## Abbreviations

cOR: Crude odds ratio
aOR: Adjusted odds ratio
CI: Confidence interval
DHS: Demographic and health survey
VIF: Variance inflation factor
SDG: Sustainable development goal
LMICs: Low- and middle-income countries
WHO: World health Organization
